# Host DNA contents in fecal metagenomics as a biomarker for intestinal diseases and effective treatment

**DOI:** 10.1186/s12864-020-6749-z

**Published:** 2020-05-11

**Authors:** Puzi Jiang, Senying Lai, Sicheng Wu, Xing-Ming Zhao, Wei-Hua Chen

**Affiliations:** 1 Key Laboratory of Molecular Biophysics of the Ministry of Education, Hubei Key Laboratory of Bioinformatics and Molecular-imaging, Center for Artificial Intelligence Biology, Department of Bioinformatics and Systems Biology, College of Life Science and Technology, Huazhong University of Science and Technology, Wuhan, 430074 Hubei China; 2 Huazhong University of Science and Technology Ezhou Industrial Technology Research Institute, Ezhou, 436044 Hubei China; 3 Institute of Science and Technology for Brain-Inspired Intelligence, Fudan University, Shanghai, 200433 China; 4 Key Laboratory of Computational Neuroscience and Brain-Inspired Intelligence, Ministry of Education, Fudan University, Shanghai, 200433 China; 5 College of Life Science, HeNan Normal University, Xinxiang, 453007 Henan China

**Keywords:** Colorectal cancer, Crohn’s disease, Gut microbiota, Diagnostic biomarkers, Treatment response, Machine learning

## Abstract

**Background:**

Compromised intestinal barrier (CIB) has been associated with many enteropathies, including colorectal cancer (CRC) and inflammatory bowel disease (IBD). We hypothesized that CIB could lead to increased host-derived contents including epithelial cells into the gut, change its physio-metabolic properties, and globally alter microbial community and metabolic capacities.

**Results:**

Consistently, we found host DNA contents (HDCs), calculated as the percentage of metagenomic sequencing reads mapped to the host genome, were significantly elevated in patients of CRC and Crohn’s disease (CD). Consistent with our hypothesis, we found that HDC correlated with microbial- and metabolic-biomarkers of these diseases, contributed significantly to machine-learning models for patient stratification and was consequently ranked as a top contributor. CD patients with treatment could partially reverse the changes of many CD-signature species over time, with reduced HDC and fecal calprotectin (FCP) levels. Strikingly, HDC showed stronger correlations with the reversing changes of the CD-related species than FCP, and contributed greatly in classifying treatment responses, suggesting that it was also a biomarker for effective treatment.

**Conclusions:**

Together, we revealed that association between HDCs and gut dysbiosis, and identified HDC as a novel biomarker from fecal metagenomics for diagnosis and effective treatment of intestinal diseases; our results also suggested that host-derived contents may have greater impact on gut microbiota than previously anticipated.

## Background

Colorectal cancer (CRC) is the 3rd most common cancer worldwide and the 2nd leading cause of cancer-related death in the United States [1, 2]; in recent years, the incidence of CRC has been increasing in young adults in major western countries [3, 4]. Similarly, Crohn’s disease (CD) is also increasing worldwide and can be attributed largely to industrial urbanization and Western life-styles [5]. As genetics could only explain limited proportions of the CRC [6, 7] and CD [8] incidences, researchers have recently linked it to environmental factors, life styles and gut microbiota dysbiosis [8–13]. By contrasting gut microbiome profiles of CRC and CD patients to that of the healthy controls, researchers have identified bacterial species that were specifically enriched in CRC [10–12, 14] and CD [13] respectively; many of the CRC-enriched species were recently found to be consistent across populations, according to two meta-analysis studies [15, 16]. In addition, microbial genes involved in various biological pathways were also enriched in the gut microbiota of CRC [10, 15, 16] and CD [13] patients. Both the differential species and pathways can be used as non-invasive markers for patient stratification [10, 11, 13, 15, 16]. These findings greatly improved our understanding on the potential roles of gut microbiota in the pathogenesis and/or development of these intestinal diseases, and implied a global alteration of the local gut environment in the patients.

The performance of gut microbiota profiling on disease diagnosis can be further improved in combination with clinical tests measuring human conditions including fecal occult blood test (FOBT) and fecal calprotectin (FCP) test [10, 17]. FOBT measures hidden blood in stool samples, indicating intestinal injury, while FCP produced by neutrophils due to activation or cell death serves as a biomarker of gut inflammation; they are markers for intestinal diseases but suffered from low specificity and sensitivity. Although clinically feasible and cost effective, it is not trivial to combine these measurements with fecal microbial profiling results. Moreover, novel non-invasive methods for CD are needed, because as a chronic and intractable gastrointestinal disease, patients with CD should be regularly monitored via colonoscopy for disease progression and/or treatment effectiveness [18, 19].

Compromised intestinal barrier (CIB) has been shown to associate with many intestinal diseases, including inflammatory bowel diseases (IBD) [20] and CRC [21, 22]. CIB could be caused by infection, lesion, and/or inflammation, manifested as a thinner mucus layer and leaky barrier, and consequently lead to increased host-derived contents from epithelial cells and blood shedding into lumen [23]. In other words, CIB could lead to increased host DNAs (also referred as to host DNA contents, HDCs) in feces of patients with intestinal diseases; the more severe the diseases, the higher HDCs. Previous researchers have detected increased human DNAs in feces from patients with intestinal diseases [24–26]. Since fecal metagenomics are obtained using whole-genome shotgun sequencing and contain unbiased survey on bacterial, viral and HDCs [13, 26], we could directly calculate the HDC as the percentage of the gut metagenomics sequencing reads mapped to the human genome (see Methods) for each fecal sample and use it as a proxy of CIB as well as a convenient approximation for FOBT and FCP tests. Furthermore, the increased host contents such as blood and human cells shedding to the intestinal tract due to CIB could alter the physio-metabolic properties of the gut environment, stimulate pro-inflammatory pathways [27] and consequently lead to global alterations in gut microbiota composition as a result of complex interplay between microbiome and host. We thus would expect that HDC, as an indicator of CIB, may also correlate with the disease-associated species and metabolic pathways.

In this study, we collected nine metagenomic datasets from two most common intestinal diseases and performed the analysis to (1) confirm that HDCs elevated in the patients signify microbial dysbiosis; (2) test whether HDC can further improve performance of machine learning models in patient stratification in combination with metagenomic profiles, and (3) evaluate the contribution of HDCs and HDC-related microbes to these models. We also analyzed the potential of HDC and microbiome for predicting treatment response to investigate the feasibility of fecal metagenomics data alone as non-invasive test.

## Results

### Increased HDCs in CRC patients

**Fig. 1:**
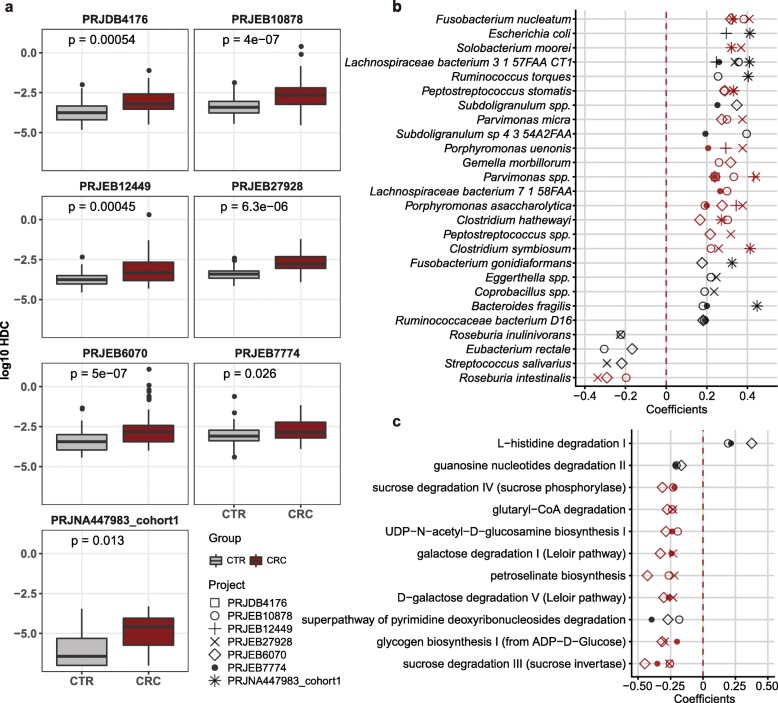
Human DNA contents (HDCs) were significantly elevated in feces of CRC patients, and correlated with microbial- and functional- biomarkers. **a**, HDCs, calculated as the percentage of gut metagenomics sequencing reads mapped to the human genome, were significantly higher in CRC (dark red box) than healthy controls (grey box) in seven recently published datasets (Wilcoxon Rank Sum Test, see Methods). **b**, Species that were significantly correlated with HDCs in two and more CRC datasets (Spearman Rank Correlation, *p*-value < 0.05, see Methods). Correlations were calculated using both CRC patients and healthy controls. Red: species with differential abundances between CRC and controls in two and more CRC datasets (Wilcoxon rank sum test, adjusted *p*-value < 0.05, see Methods). These species were referred as to HDC-species in this study. **c**, Metabolic pathways that were significantly correlated with HDCs in three and more CRC datasets. Correlations were calculated using both CRC patients and healthy controls. Red: pathways with differential abundances between CRC and controls in two and more CRC datasets (Wilcoxon rank sum test, adjusted *p*-value < 0.05, see Methods). These species were referred as to HDC-pathways in this study

We first focused on CRC. As expected, we found that HDCs were significantly higher in feces of CRC patients than that of the healthy controls in all seven datasets (Fig. 1a, Additional file 1: Table S1 and Additional file 2: Table S2). We then identified in total 26 species that were significantly correlated with HDCs in at least two datasets (Spearman Rank Correlation, *p*-value < 0.05, Fig. 1b; see Methods and Additional file 3: Table S3) and referred them as HDC-species below. We also identified species that showed significantly differential abundances between case and controls in at least two CRC datasets (adjusted *p*-value < 0.05, see Methods) and referred them as Dif-species (also known as CRC-signature species). Interestingly, we found half of the HDC-species (13 out of 26) overlap with the CRC Dif-species, including 12 CRC-enriched ones (Fig. 1b) such as *Fusobacterium nucleatum, Bacteroides fragilis* and *Peptostreptococcus stomatis*, which were found in two recent meta-analyses of CRC [15, 16]. Microbial colonization varies along the colon, partly because of thickness of mucous layer. Previous studies showed the *B. fragilis* with the capability of glycoproteins degradation and toxin production could penetrate the protective mucous layer, suggesting the bacteria accelerate the injury of gut barrier, trigger inflammation and induce tumorigenesis [28–30].

We also identified 40 HDC-correlated metabolic pathways in at least two datasets (referred as to HDC-pathways, see Additional file 4: Table S4); among which, 16 were identified as metabolic pathways with differential abundances between patients and controls in at least two datasets (referred as to Dif-pathways, see Methods). Most of the HDC-pathways that decreased in at least three datasets were related to carbohydrate degradation for production of energy and short-chain fatty acids, such as D-galactose degradation and sucrose degradation (Fig. 1c) [31]. In addition, HDC negatively correlated with the degradation pathways of several monosaccharides and monosaccharide derivatives, including fucose, mannose, galactose and UDP-N-acetyl-D-glucosamine (Additional file 4: Table S4), which are known building blocks of gut mucus glycans; these results indicated decreased concentrations of the monosaccharides and derivatives, further confirming that the intestinal barrier is compromised [30].

Together, our results suggested that CIB, as indicated by HDCs that can be directly quantified from gut metagenomics data, maintained a relationship with gut microbiota dysbiosis both in taxonomic and functional levels.

### Combination of HDC and microbiome contributed significantly to patient stratification

**Fig. 2:**
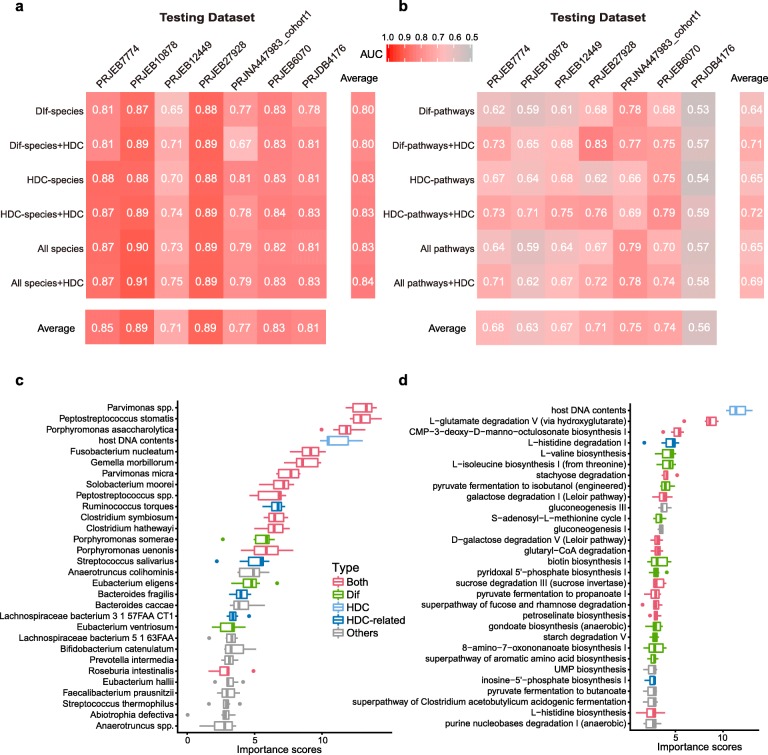
HDC and correlated species and metabolic functions contribute significantly to patient stratification in LODO analysis in CRC. **a**, Predictive performances as AUC values obtained using LODO analysis by training the models on the species abundances. The AUC values were averaged from repeated results of 10-fold validation analysis. The labels of y-axis mean the features used for building models. Dif-species: species whose abundances are significantly different between CRC and controls in at least two datasets (Wilcoxon Rank Sum Test, see Methods); HDC-species: HDC-correlated species in at least two datasets; see Methods for details. All-species: the overall species. **b**, AUC values obtained using LODO analysis by training the models on the metabolic pathway abundances. The labels of y-axis mean the features used for building models. Dif-pathways: pathways whose abundances are significantly different between CRC and controls in at least two datasets (Wilcoxon Rank Sum Test, see Methods); HDC-pathways: HDC-correlated pathways; see Methods for details. All-pathways: the overall pathways. **c-d**, Ranking of feature importance in the HDC + All-species model **c** and HDC + All-pathways model **d**. The models were trained by using HDC values and relative abundances of all species/pathways as input. The importance scores were reported by the LODO models. The features were ranked according to the median importance scores from 100 repeated results of 10-fold cross-validation analysis. Dif: species/pathways whose abundances are significantly different between CRC and controls in at least two datasets (see Methods); HDC-related: species/pathways correlated with HDC in at least two datasets (see Methods); Both: differential species/pathways that was also correlated with HDC; HDC: host DNA contents; Other: species/pathways that were neither HDC-related nor differential

We next tested if HDC-species and HDC-pathways could contribute to patient stratification in CRC. Similar to Wirbel et al [15] and Thomas et al [16], we performed a leave-one-dataset-out (LODO) analysis [32] in which Random forest classifiers were trained on the combined datasets of all but one, and tested on the one that was left-out; we did this for each dataset in turn. As shown in Fig. 2a and b, for models trained using species and pathways abundances, including HDCs could improve prediction performance. More importantly, HDC was ranked as a top feature, i.e. the 4th and 1st in the taxonomic (Fig. 2c) and functional (Fig. 2d) models, respectively. Interestingly, both HDC-related models performed better than models based on Dif-species and Dif-pathways, even though overlap existed in the taxonomic and functional features (Fig. 2a, b). These results indicated the HDC-correlated features could contribute substantially to patient stratification and disease diagnosis (Fig. 2).

### Similar results were found in CD

We then checked if similar results could be found in CD. A previous study reported elevated fecal HDCs in pediatric CD patients as compared with healthy controls [13]; the authors used quantitative polymerase chain reaction (QPCR) method to quantify HDCs by targeting human beta-tubulin coding-sequences. The authors also calculated HDCs from the metagenomics data and reported that the QPCR results were positively correlated with metagenomics-data-derived HDC values (r = 0.81 Pearson’s correlation, *p* = 9.3 × 10^− 11^; see ref. [13]). We re-calculated the HDCs using our methods and found they were highly correlated with theirs (r = 0.978 Pearson’s correlation, *p* < 2.2e-16; Additional file 5: Table S5). These results further validated the reliability and accuracy of metagenomics-derived HDCs.

**Fig. 3:**
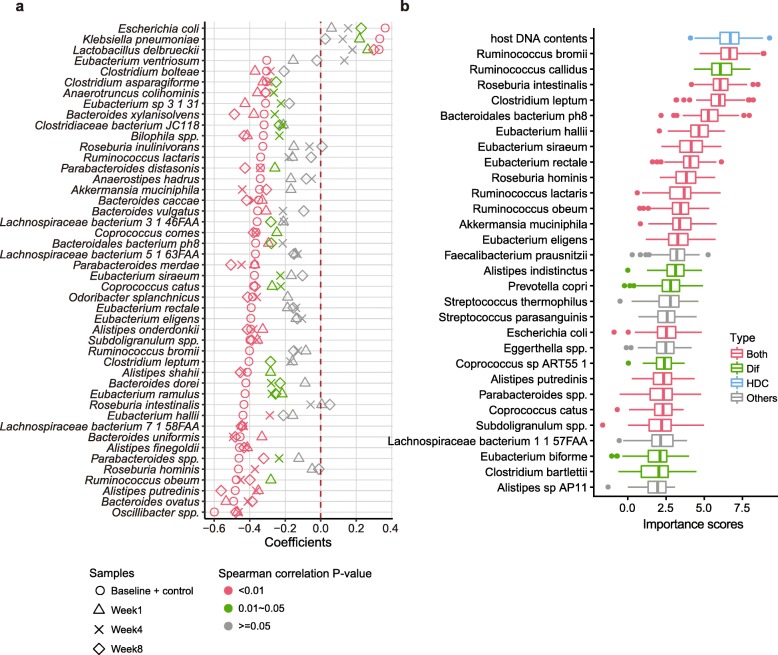
HDC was also elevated in CD, correlated with differential species and contributed significantly to patient stratification. **a,** Species that were correlated with HDCs in the group of healthy controls and untreated patients (Baseline + Control, Spearman correlation, *p*-value < 0.001). Also plotted are the correlation coefficients between HDCs and species abundances in patients at three time-points after they were treated (Week1, Week4 and Week8). Correlation coefficients were color-coded according to their significance levels. **b,** Ranking of feature importance in the HDC + All-species model. The models were trained by using HDC values and relative abundances of all species as input; only the data of the healthy controls and untreated patients were used. The importance scores were reported by the Random forest models. The features were ranked according to the median importance scores from 100 repeated results of cross-validation analysis (see Methods). Dif: species whose abundances are significantly different between untreated CD and controls (see Methods); Both: differential species that was also correlated with HDC; HDC: host DNA contents; Other: species that were neither HDC-related nor differential

We identified 46 HDC-species (Control+Baseline group, Spearman correlation, *P*-value < 0.001), most of which (31 out of 47) overlapped with the Dif-species of CD that showed significant abundance changes between healthy controls and untreated patients (Control+Baseline group, Wilcoxon rank sum test, adjusted *p*-value < 0.05, Fig. 3a, Additional file 6: Table S6 and Additional file 7: Table S7). *Akkermansia muciniphila* and *Bacteroides caccae* as mucus-degrading commensal species, were expectedly reduced with increasing HDCs, because impaired gut was insufficient to secrete mucus [33]. Another control-enriched bacterial marker, *Eubacterium ventriosum*, was previously identified to be negatively associated with fundamental components of eukaryotic cell membranes [34]. Similarly, differential pathways partly overlapped with HDC related pathways, including those involved in carbohydrate, protein and glycogen metabolism, the decreased abundances of which were known to associated with nutrient deficiency and dysfunction of intestine (Additional file 8: Table S8 and Additional file 9: Table S9) [31, 35, 36].

We also built random forest classifiers using species and pathways abundances for CD and did 10 times repeated 10-fold cross-validation. Similar to CRC, we found that adding HDC to the input data could improve prediction performance (AUC increased from 0.94 to 0.95 based on species profile; increased from 0.90 to 0.92 based on pathways profile; Additional file 10: Fig. S1); similar to CRC, we found that HDC was ranked as a top important feature (1st in this case), and majority of top 10 features were HDC-species (Fig. 3b). Interestingly, although overlapped significantly, these species are quite different from those in CRC (Additional file 11: Table S10) in terms of their changes and importance in patient stratification (Fig. 3b), likely due to differences of disease localizations and microenvironments: CD commonly occurred in the terminal part of ileum and present an inflammatory habitat for microbes, while CRC appearing as tumor microenvironment occurred in the colorectum [37, 38]. Nonetheless, it appears that elevated HDC is a common feature of intestinal diseases, while different diseases can be distinguished by their different gut dysbiosis profiles.

### HDC and related dysbiosis signified clinical treatment outcomes

**Fig. 4:**
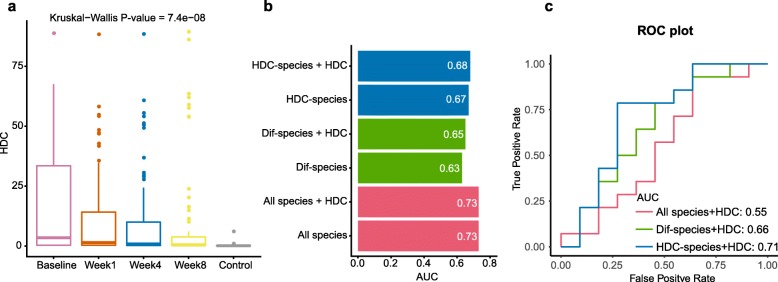
HDCs were reduced during treatment, and could improve the performance of machine learning models in predicting treatment response of CD patients. **a**, HDCs were significantly reduced along treatment intervention. **b**, Predictive results as AUC values obtained from 10-time repeated 10-fold cross-validation models for classifying treatment response. The labels of y-axis mean the features used for building models. HDC-species: HDC-correlated species in untreated patients and controls (see Methods for details); Dif-species: species whose abundances are significantly different between untreated patients and controls (Wilcoxon Rank Sum Test, see Methods); All-species: the overall species. **c**, External validation of models based on HDC and species showing in Fig. 4**b**. Accuracies were displayed as ROC plot, in which x axis is false positive rate, y axis is true positive rate, and AUC is the area under the curve

The CD patients we analyzed were treated with diet intervention or anti-TNF antibodies; the outcomes were evaluated with fecal metagenomics sequencing at week 1, 4 and 8 after the interventions [13]. We found that the HDCs were significantly decreased over time (Fig. 4a). As expected, HDC correlates significantly with FCP (Pearson’s correlation = 0.498, *p* < 2.2e-16, Additional file 12: Fig. S2), a clinical indicator of intestinal inflammation released by neutrophils. However, concentrations of FCP were only associated with 3 CD Dif-species, indicating that HDC is a better biomarker related with dysbiosis than FCP. Strikingly, we found 23 of the HDC-species in CD showed coordinated changes with HDC, i.e. species that were positively (negatively) correlated with HDC in the Control+Baseline group decreased (increased) with the decreasing HDCs (Kruskal-Wallis rank sum test, adjusted *p*-value < 0.05, Additional file 13: Fig. S3), suggesting that the intervention that reduced fecal HDCs could globally reverse the gut dysbiosis in a species-specific manner. Such a conclusion was further supported by the observation that the correlations between HDC and some of the species were consistent in the Control+Baseline, Week1, Week4 and Week8 groups (Fig. 3a).

We then investigated the effects of classifiers based on HDC and gut microbiome in predicting response to CD therapy (see Methods). As we expected, including HDC to the models could improve performances (Fig. 4b, Additional file 14: Fig. S4); again, we found that models based on HDC-species performed better than models based on Dif-species. These results suggested we need reform the previous thinking that considers only changed species as biomarkers of patients, because there were some species whose alterations did not reach the significance threshold (e.g. fdr < 0.05) but had a tendency. Besides, according to accuracies of classifiers built on pathways, we hypothesized that the microbial functional network didn’t change a lot during treatment, even if the conditions of the patients were improved over time (Additional file 14: Fig. S4). To confirm our hypothesis above, we collected another metagenomics dataset of CD patients for external validation. Interestingly, models built on HDC and HDC-species performed better (AUC = 0.71, Fig. 4c) than other models (AUCs≤0.66) (Additional file 15: Table S11). Most of the top features of HDC related classifier are consistent with foregoing results that several HDC-species tended to recover when patients were under treatment (Additional file 16: Fig. S5). The performance of the classifiers confirmed our inference that HDC related features (i.e. HDC-species) had the potential to be signatures in classifying therapeutic responses (Fig. 4b, Additional file 15: Table S11).

## Discussion

In this study, we showed that HDCs in fecal metagenomic data were significantly elevated in patients of intestinal diseases, and thus could be used as a quantitative indicator for CIB. CIB can increase the host-derived contents including epithelial cells and/or blood to be shed into intestinal lumen, alter the local gut environment and facilitate gut microbiota dysbiosis in view of the reciprocal relationship between gut microbiota and the host [39, 40]. As we expected, HDCs as a proxy of CIB, showed a higher abundance in feaces of patients, correlated significantly with many disease-altered species and metabolic pathways in CRC and CD, and can also be used as a quantitative indicator of gut microbiota dysbiosis.

Age, gender and BMI (body-mass index) are known confounding factors of the taxonomic profiles of fecal metagenomic data. To check if the differential HDCs could also be attributed to them, we tested if these factors were well matched between the cases and controls within the projects. Six out of the seven CRC datasets showed well-matched gender, age, and BMI profiles (Additional file 17: Table S12). For the remaining dataset, we applied a generalized linear modeling function (glm) to control for the three confounders; we found that the HDCs were still significantly higher in cases than in controls (Additional file 18: Table S13). For the CD dataset, the meta-data were not available. However, according to the related publication [13], the authors performed similar statistical analysis and found no significant differences on gender and age between patients and controls. We thus believe that the elevated HDCs were not the results of biased sample characteristics.

We further tested if biogeographic ancestry had impacts on our analysis. We analyzed the dataset that consisting of samples from two countries (PRJEB6070), and found that there was no difference in microbial alpha diversity between Germany and France (Wilcoxon rank sum test, CTR: *p*-value = 0.059, CRC: *p*-value = 0.16). We also did cross-project comparison, and found that all projects tended to have similar levels of HDCs in their cases and controls respectively, although each project focused on samples of different countries from others (Fig. 1a). These results suggested our results were not influenced by biogeographic ancestry of the samples.

So far researchers have mostly focused on the potential of gut microbes as non-invasive biomarkers solely, or together with an extra test indicating CIB, such as FOBT and FCP, which are fast but low-sensitive and low-specific. Our results suggested that CIB could be quantified as HDCs from metagenomics data, that were directly proportional with concentrations of FCP. As we have expected, including HDC as an additional feature, we obtained better machine learning models in patient stratification in both CRC and CD.

We also showed that HDC can be used as an indicator for effective treatment. HDCs were reduced significantly during diet or drug intervention in CD patients, which were accompanied by the recovery of many CD-related species. FCP correlated with only a few of the species, suggesting that HDC can be a better indicator for the global recovery of gut dysbiosis. Again, machine learning models including HDC achieved better performance in predicting treatment responses in patients.

Surprisingly, we found that the overall model performance based on HDC-correlated features was better than the differential features (i.e. taxonomic or functional features that showed significant differences between patients and healthy controls), although they overlapped significantly. These results suggest that the perturbation of important species could also contribute to disease development, even though the differences were not statistically significant. In addition, we found there were some biases in distribution of HDC-related features, which were mainly supplied by samples from Chinese, Germany and Australian and rarely contributed by Japanese and USA (Additional file 19: Table S14). However, the LODO results showed that the HDC-related species/pathways based models worked well on each projects (mean ± SD of AUC: 0.83 ± 0.07 in HDC-species model, 0.65 ± 0.07 in HDC-pathways model, see Fig. 2), suggesting HDC-related species have the potential to be common features. These results also highlighted the robustness of meta-analysis.

In silico removal of host DNAs from metagenomics data is a recommended procedure [41], in part due to the considerations of contamination. Our results indicate that the metagenomics data can validate itself by looking at the correlated changes in HDC and related gut microbial species: a sudden increase in HDC without matching alterations in related bacteria is a strong indication of contamination. This line of reasoning can be applied to any host-produced molecules identified from feces, such as DNA, RNAs, proteins, metabolites and even cells, and would pave the way for extracting more host related information directly from fecal samples using multi-omics techniques and making use of them without worrying too much about contamination. As we have shown in this study, host related information directly extracted from fecal samples is reliable and useful.

In summary, we found that CIB as indicated by elevated human DNA contents (HDCs) in feces, is a common feature of intestinal diseases; HDC could be a promising biomarker for intestinal diseases because it signified the abundances changes of most of disease-related species, ranked as the top contributor to machine learning models for patient stratification and treatment response.

## Conclusions

We identified that intestinal injury, manifesting as increased HDCs s in faeces were associated with gut microbiome dysbiosis, and microbial community could be reversed during treatment along with reduced HDCs. Including HDCs as an additional feature to metagenomics-based classifiers could improve their performance in patient stratification. Since both HDCs and the taxonomic and metabolic profiles could be calculated from metagenomic sequencing data, our study further supported the fecal metagenomics as a means for non-invasive diagnosis and even assessment of therapeutic response of intestinal diseases.

## Methods

### Metagenomics data analysis

A total of 354 CRC patients, 110 CD patients and 382 controls from nine fecal metagenomics datasets were included in this study for classifier construction and external validation. Raw sequencing reads and metadata of the seven human CRC metagenomics datasets were obtained from European Nucleotide Archive (ENA) under the following ENA project identifiers: PRJEB10878 [11], PRJEB27928 [15], PRJEB7774 [12], PRJEB12449 [14], and PRJEB6070 [10], PRJNA447983 [16] (cohort 1), and PRJDB4176 [15]. Raw sequencing reads and metadata of the CD metagenomics dataset were obtained from NCBI SRA database under SRA ID: SRP057027 [13] for model construction and PRJNA384246 for external validation [19]. More details, including the nationality and age of these subjects, can be found in Additional file 1: Table S1.

To remove adapters and low quality of bases, raw reads were filtered and trimmed by Trimmomatic [42] v3.6, using the Truseq3 adapter files and option with MINLEN cutoff 50. To estimate the human DNA contents (HDC) in metagenomics sequencing reads, the remaining reads (clean reads) were aligned to the human reference genome (hg19) using bowtie2 [43] (version 2.3.4.3); the HDC of a sample was calculated as the percentage of mapped reads out of total clean reads in the sample. The human DNA contents measured by quantitative PCR (QPCR) results in CD dataset were obtained from the corresponding publication by Lewis and colleagues [13]. Reads mapped to the human genome were removed before subsequent analyses. Taxonomic abundances of all metagenomic samples were quantified using MetaPhlAn2 [44]. HUMAnN2 [45] was used to calculate relative pathway abundances via mapping reads to ChocoPhlAn database and full UniRef90 database.

In each project except PRJNA384246 which was for external validation, to remove noise, species with max abundance < 0.1% in all samples as well as species whose average abundance across all samples below 0.01% were removed from further analyses. Similarly, pathways with zero value in at least 15% samples and with maximum relative abundances less than 1× 10^− 6^ in all samples of a project were also removed. The abundance data were then loaded into R and analyzed (https://www.r-project.org; version 3.5.1).

### Statistical analyses

Wilcoxon Rank Sum Test was used to detect significant between-group differences in relative abundances of taxonomic- and pathway- features; features with adjusted *P*-value < 0.05 in at least two datasets of CRC were deemed significant; Similarly, differential features with adjusted *P*-value < 0.05 between controls and untreated patients (marked as baseline) in CD dataset were deemed significant. Spearman correlation was used to find HDC correlated species and pathways, features with *P*-value < 0.05 in at least two datasets of CRC were selected as significantly correlated features. For CD dataset, HDC related species and pathways with *P*-value < 0.001 were identified in controls and untreated patients using Spearman correlation. Besides, for determining the relationships between HDCs and species during the treatment, those identified HDC-related species were used to compute their spearman correlations with HDCs in each treatment period (Week1, week 4 and week8). Also we used Kruskal-wallis test to examine which HDC-related species decreased significantly during therapy.

### Classifiers construction

The randomForest package in R was used to build mathematic classification models (classifiers) that are capable of distinguishing patients and tumor-free participants, extract features that can be used to discriminate different phenotype groups and calculate feature importance scores. The createMultiFolds algorithm of caret package was used to split dataset into 10 folds repeatedly to avoid biases due to simply one split.

For the CRC data, a so-called LODO analysis was also performed in order to evaluation cross-study performance of the obtained classifiers. In LODO analyses, all datasets except the one used for model testing were pooled as a training dataset which would be implemented the within-dataset 10-fold cross-validation; LODO was performed for each dataset in turn and were repeated 10 times, for all the seven CRC datasets. The LODO training dataset prediction accuracy was measured through 10 times repeated 10-fold cross-validation.

For the CD data, 10 times repeated 10-fold cross-validation was used to assess the within-dataset accuracy of the resulting classifiers. We first utilized the data of controls and baseline for building classifiers to distinguish CD patients from controls. Then we constructed classifiers for predicting treatment response, only considering patients with complete longitudinal records whose FCP over 250 μg/g at baseline [13]. We defined response to therapy as a decrease in FCP to ≤250 μg/g in a given period. For external validation, the information of therapeutic response was obtained from original article, which estimated clinical response according to HBI and SCCAI [19].

## Supplementary information

**Additional file 1: Table S1.** A list of CRC and CD projects used in this study and the numbers of controls and cases.

**Additional file 2: Table S2.** Metadata of participants in CRC projects and their HDC%. HDC means the human DNA content.

**Additional file 3: Table S3.** Species that correlated with HDC in more than two CRC datasets statistically. Species whose Spearman *p*-value < 0.05 in at least two projects were deemed as HDC correlated species. The column overlap = “1” means that the species is a signature feature whose abundance were significantly different in patients when compared with controls.

**Additional file 4: Table S4.** Pathways that correlated with HDC in more than two CRC datasets statistically. Pathways whose Spearman p-value < 0.05 in at least two projects were deemed as HDC correlated Pathways. The column overlap = “1” means that the pathway is a signature feature whose abundance were significantly different in patients when compared with controls.

**Additional file 5: Table S5.** A list of samples of the CD project (SRP057027) and their HDC% produced by two ways. Our HDC% were generated using Bowtie2, while Lewis’s HDC% were generated using BMtagger (pmid:26468751).

**Additional file 6: Table S6.** Species that correlated with HDCs in untreated patients and controls from CD project (*P*-value < 0.001). We calculated Spearman correlation between HDC and HDC-related species in each stage of patients and controls.

**Additional file 7: Table S7.** Differential species in the Control+Baseline group of CD (Adjusted P-value < 0.05). The column overlap = “1” means that the species were correlated with HDC (Spearman correlation P-value < 0.001, see Table S6).

**Additional file 8: Table S8.** Pathways that correlated with HDCs in untreated patients and controls from CD project (P-value < 0.001). We calculated Spearman correlation between HDC and HDC-related pathways in each stage of patients and controls.

**Additional file 9: Table S9.** Differential pathways in the Control+Baseline group of CD (Adjusted P-value < 0.05). The column overlap = “1” means that the pathways were correlated with HDC (Spearman correlation P-value < 0.001, see Table S8).

**Additional file 10: Figure S1.** AUC of random forest classifiers based on species/pathways profiles (SRP057027) for predicting untreated CD patients from controls. The labels of y-axis mean the features used for building models. Dif-species/pathways: species/pathways whose abundances are significantly different between untreated CD patients and controls (see Methods); HDC-species/pathways: species/pathways correlated with HDC (see Methods); all-species/pathways: the overall species/pathways.

**Additional file 11: Table S10.** Overlapped features of the top 30 important features between CD and CRC. The columns “Importance_in_CRC” and “Importance_in_CD” mean the median importance scores of the top 30 features in the CRC models and CD models, respectively. The columns “ranking_in_CRC” and “ranking_in_CD” mean the importance degrees of the shared features in the CRC models and CD models separately.

**Additional file 12: Figure S2.** Pearson correlation between HDC and FCP in CD dataset (SRP057027).

**Additional file 13: Figure S3.** Distributions of consistently HDC-correlated species in controls and patients with complete longitudinal treatment of CD dataset (SRP057027). Y-axis is log10 transformed relative abundances.

**Additional file 14: Figure S4.** AUC of random forest classifiers based on pathways profiles (SRP057027) for predicting treatment response. The labels of y-axis mean the features used for building models. HDC-related pathways: pathways correlated with HDC (see Methods); Dif-pathways: pathways whose abundances are significantly different between untreated CD patients and controls (see Methods); all-pathways: the overall pathways.

**Additional file 15: Table S11.** AUC of classifiers based on species/pathways profiles (SRP057027) when predicting treatment response of external dataset (PRJNA384246). Features column means which type of features used for constructing random forest model.

**Additional file 16: Figure S5.** Ranking of feature importance in the HDC + HDC-related species model for predicting treatment response. The models were trained by using HDC values and relative abundances of HDC-related species as input; only the data of the patients with complete longitudinal treatment were used. The importance scores were reported by the Random forest models. The features were ranked according to the median importance scores from 100 repeated results of cross-validation analysis (see Methods). Both: HDC-related species whose abundances were differential significantly between untreated CD patients and controls; HDC: host DNA contents; HDC-related: species that were correlated with HDC. Those species marked a star in front of the name were the consistent HDC-related species shown in Fig. S3.

**Additional file 17: Table S12.** Characteristics of controls and CRC patients in seven CRC projects.

**Additional file 18: Table S13.** Generalized linear model based on the samples from PRJEB27928 to identify the significant predictors associated with CRC.

**Additional file 19: Table S14.** The contribution of each dataset on identification of HDC-related species and HDC-related pathways. The column “count_HDCrelated_species” and “count_HDCrelated_pathways” mean the count of identified HDC-related species and HDC-related pathways respectively in the corresponding dataset.

## Data Availability

All R codes are available at https://github.com/evolgeniusteam/HumanDNAContents_in_gut_metagenomics; also available are instructions for users to reproduced all our analyses, including figures, supplementary figures and statistics. The datasets generated during the current study are not publicly available due to file size but are available from the corresponding author W.H.C. on reasonable request.

## Abbreviations

CIB: Compromised intestinal barrier
HDC: Host DNA content
CRC: Colorectal cancer
CD: Crohn’s disease
IBD: Inflammatory bowel disease
FCP: Fecal calprotectin
LODO: Leave-one-dataset-out
QPCR: Quantitative polymerase chain reaction
AUC: Area under the receiver-operating characteristics curve
BMI: Body-mass index
