# Supplementary figures and images for: Host DNA contents in fecal metagenomics as a biomarker for intestinal diseases and effective treatment
Source: BMC Genomics. 2020 May 11;21:348. doi: 10.1186/s12864-020-6749-z (PMC7216530; doi:10.1186/s12864-020-6749-z)

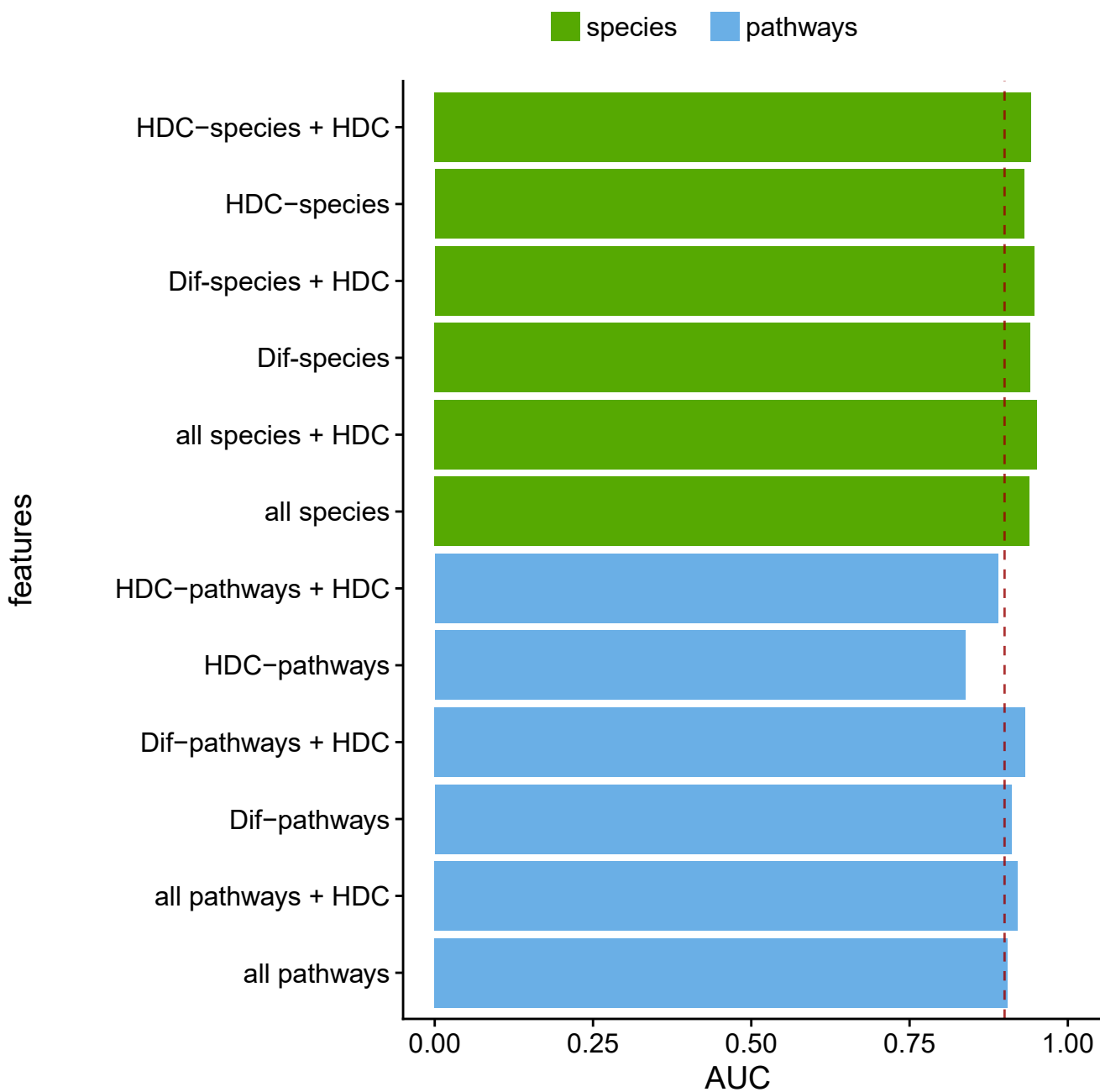

Supplement: Supplementary file 10 — Additional file 10: Figure S1. AUC of random forest classifiers based on species/pathways profiles (SRP057027) for predicting untreated CD patients from controls. The labels of y-axis mean the features used for building models. Dif-species/pathways: species/pathways whose abundances are significantly different between untreated CD patients and controls (see Methods); HDC-species/pathways: species/pathways correlated with HDC (see Methods); all-species/pathways: the overall species/pathways. [file 12864_2020_6749_MOESM10_ESM.pdf]

Pearson coefficient = 0.498, P-value < 2.2e-16

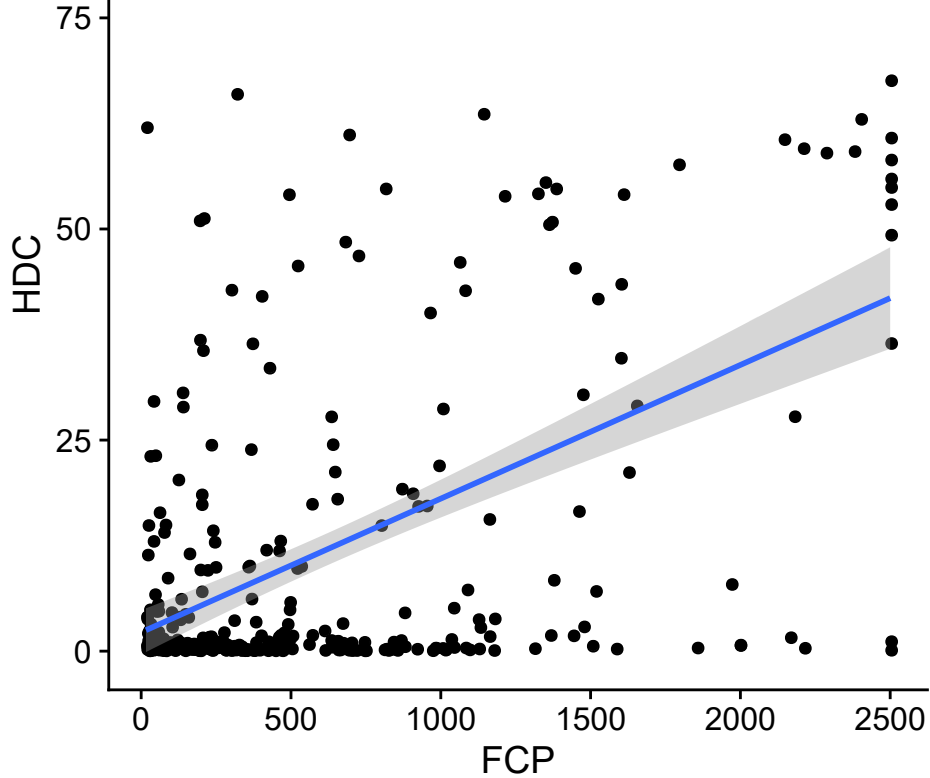

Supplement: Supplementary file 12 — Additional file 12: Figure S2. Pearson correlation between HDC and FCP in CD dataset (SRP057027). [file 12864_2020_6749_MOESM12_ESM.pdf]

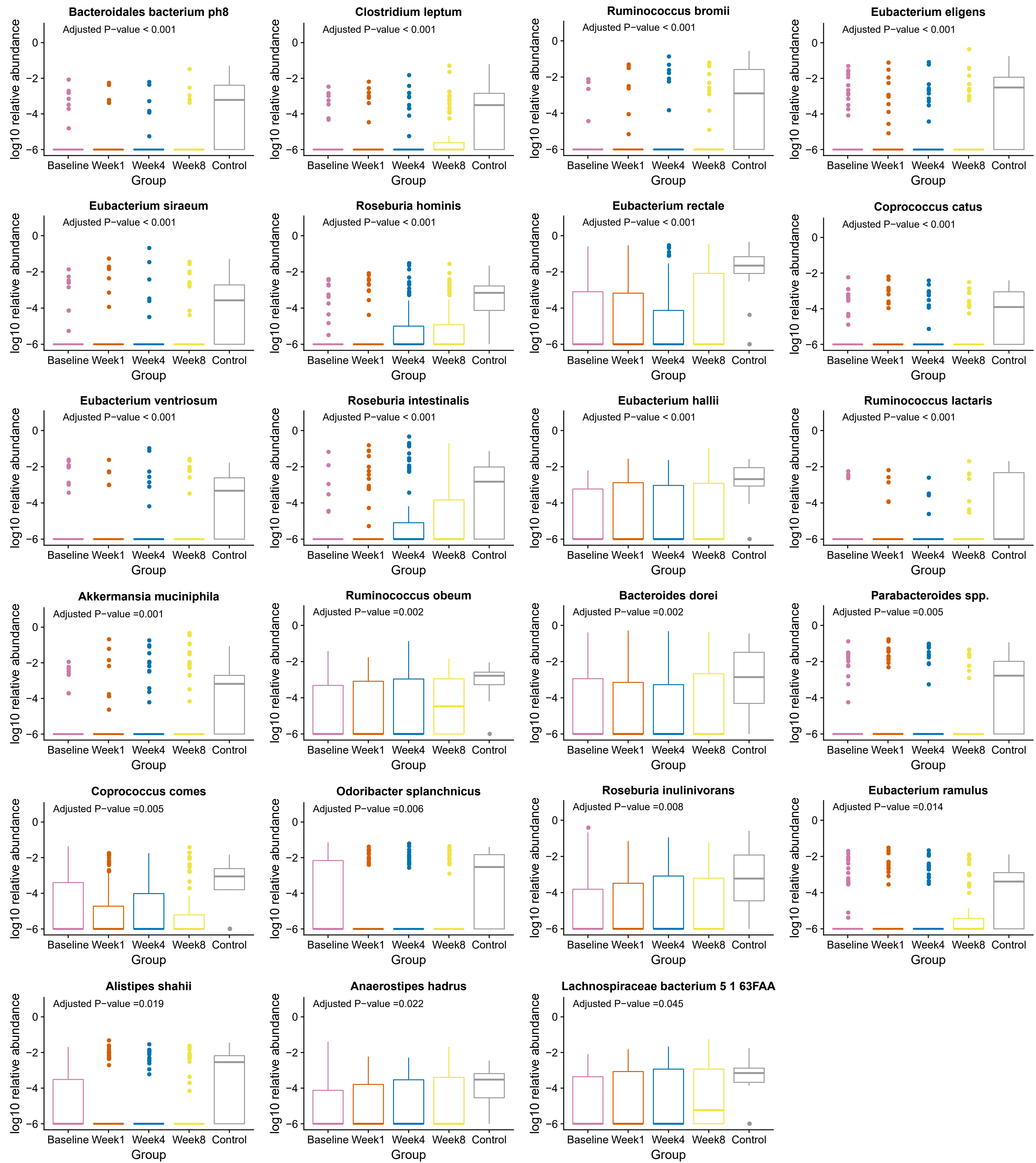

Supplement: Supplementary file 13 — Additional file 13: Figure S3. Distributions of consistently HDC-correlated species in controls and patients with complete longitudinal treatment of CD dataset (SRP057027). Y-axis is log10 transformed relative abundances. [file 12864_2020_6749_MOESM13_ESM.pdf]

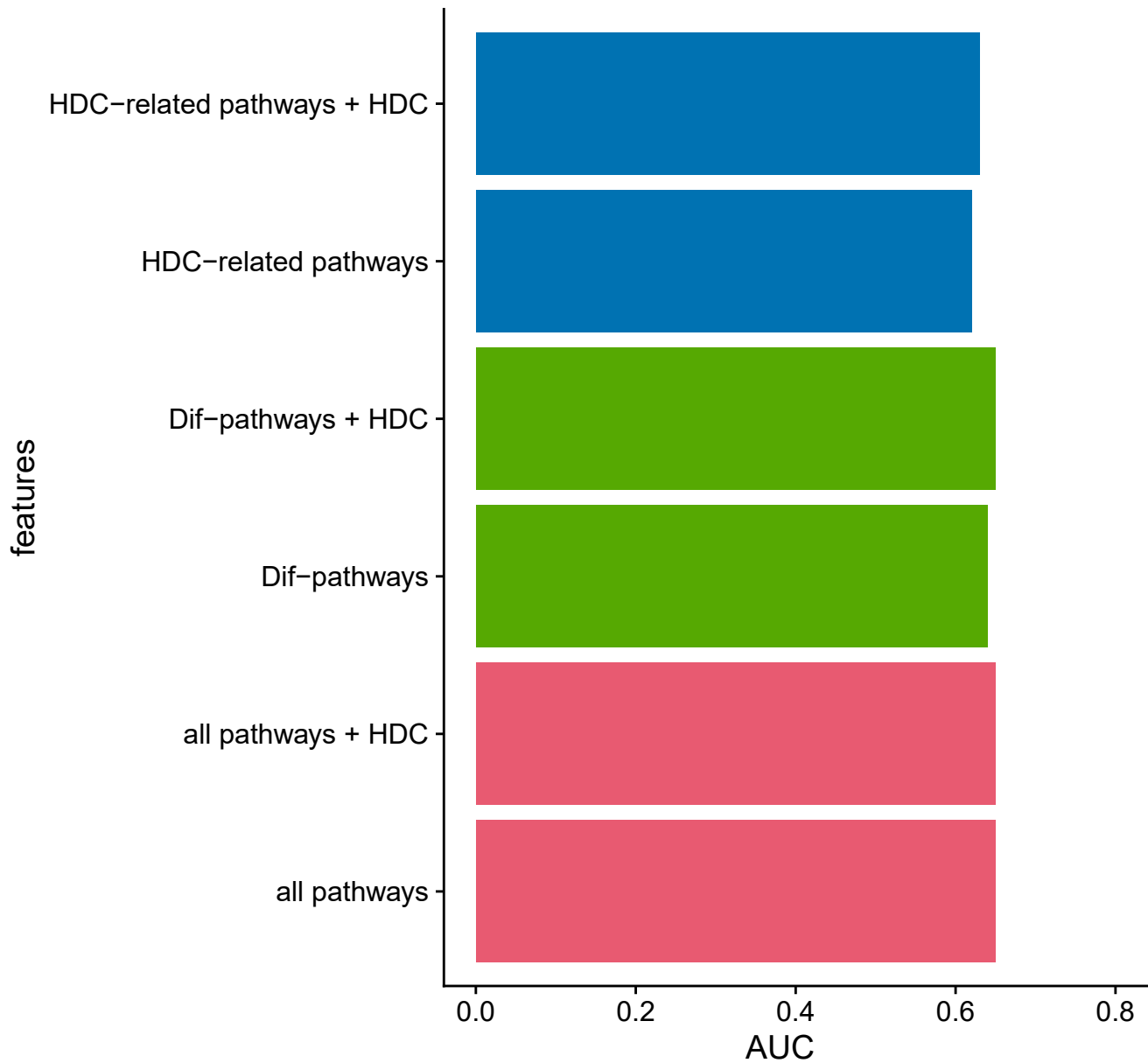

Supplement: Supplementary file 14 — Additional file 14: Figure S4. AUC of random forest classifiers based on pathways profiles (SRP057027) for predicting treatment response. The labels of y-axis mean the features used for building models. HDC-related pathways: pathways correlated with HDC (see Methods); Dif-pathways: pathways whose abundances are significantly different between untreated CD patients and controls (see Methods); all-pathways: the overall pathways. [file 12864_2020_6749_MOESM14_ESM.pdf]

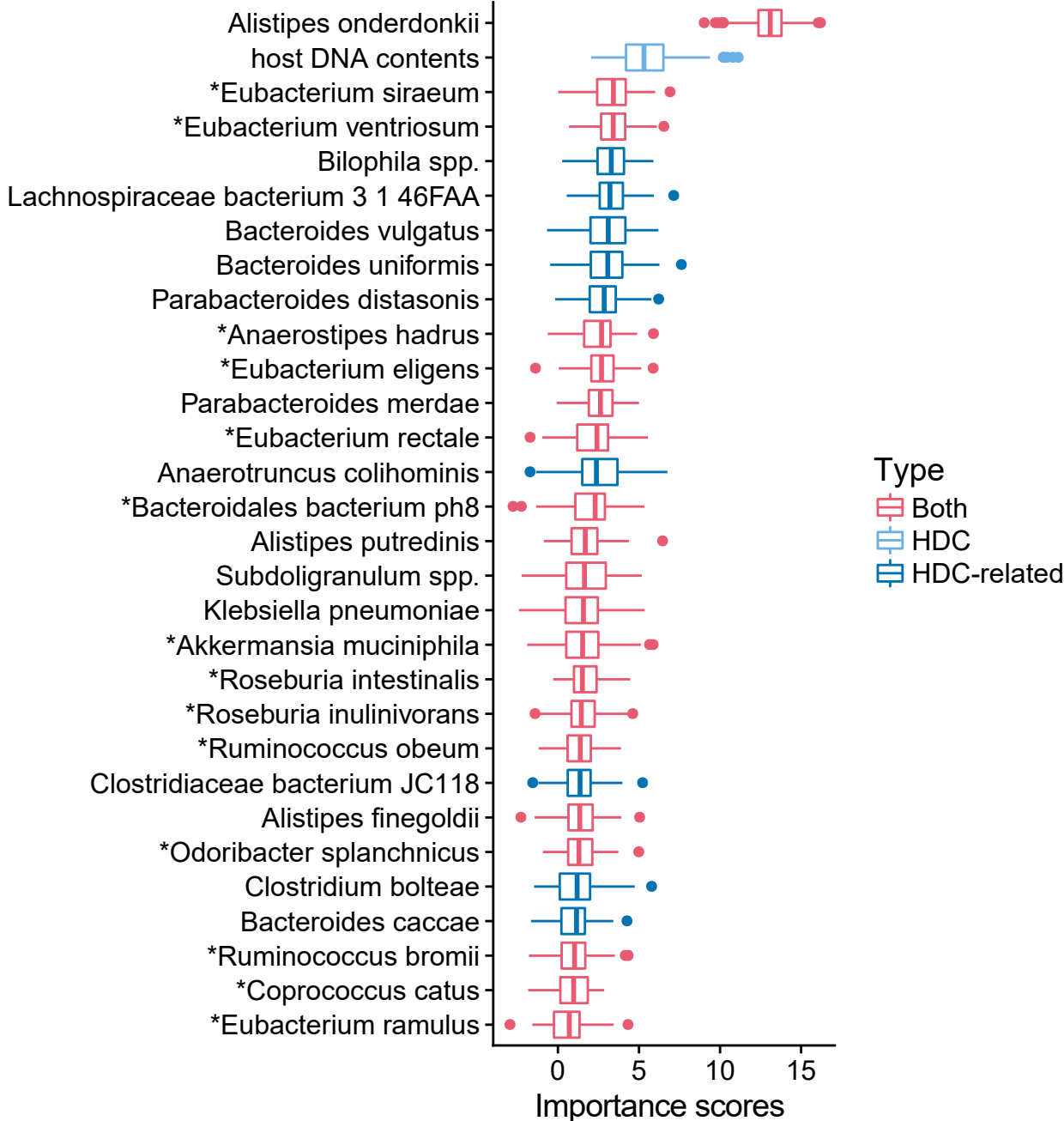

Supplement: Supplementary file 16 — Additional file 16: Figure S5. Ranking of feature importance in the HDC + HDC-related species model for predicting treatment response. The models were trained by using HDC values and relative abundances of HDC-related species as input; only the data of the patients with complete longitudinal treatment were used. The importance scores were reported by the Random forest models. The features were ranked according to the median importance scores from 100 repeated results of cross-validation analysis (see Methods). Both: HDC-related species whose abundances were differential significantly between untreated CD patients and controls; HDC: host DNA contents; HDC-related: species that were correlated with HDC. Those species marked a star in front of the name were the consistent HDC-related species shown in Fig. S3. [file 12864_2020_6749_MOESM16_ESM.pdf]
